# Inhibition of autophagy enhances synergistic effects of Salidroside and anti-tumor agents against colorectal cancer

**DOI:** 10.1186/s12906-017-2046-z

**Published:** 2017-12-16

**Authors:** Hai Li, Chen Chen

**Affiliations:** 1 Department of Geriatrics, Tongji Hospital, Tongji Medical College, Huazhong University of Science and Technology, Wuhan, China; 2 Department of Breast and Thyroid Surgery, Union Hospital, Tongji Medical College, Huazhong University of Science and Technology, Wuhan, China

**Keywords:** Salidroside, Autophagy, AMPK signaling, Anti-tumor agents, Colorectal cancer

## Abstract

**Background:**

Various plant extracts have been suggested to be used as auxiliary agents in chemotherapy considering their anti-proliferative effect on cancer cells. However, recent reports reveal that plant extracts may function as inducers of autophagy of cancer cells. In general, autophagy confers survival advantage for cells responding to stress conditions, thus representing an important mechanism for chemo-resistance. This study was aimed to investigate the effectiveness of combined use of Salidroside (Sal, a phenylpropanoid glycosides from *Rhodiola rosea L*) with anti-tumor agents against colorectal cancer (CRC) cells, and moreover to evaluate the potential role of autophagy in the combined therapy.

**Methods:**

CRC cells, HCT-116, were incubated with Sal alone or in combination with conventional chemotherapy agents including oxaliplatin (OXA), 5-fluorouracil (5-FU) and Doxorubicin (ADM). Cell proliferative characteristics were evaluated by cell viability and apoptosis rate. The protein expression was assessed by Immunofluorescent and Western blot assays.

**Results:**

Sal, alone or in combination with anti-tumor agents, increased expression of autophagic biomarkers, including LC3B and Becline-1, suggesting an autophagy induction. Except for the up-regulation of p-AMPK, p-mTOR, p-NF-κB (p65), TGF-β, p-JAK2 and p-STAT3 were down-regulated by Sal. Because autophagy is positively correlated with the activation of AMPK**/**mTOR, NF-κB, TGFβ1 and JAK2/STAT3 cascades, the autophagy induced by Sal may associate with AMPK activation. Indeed, blockage of AMPK signaling via Compound C or AMPK knockdown inhibited the autophagy. The blockage of AMPK signaling or a direct inhibition of autophagy via 3-MA increased effectiveness of combined use of Sal with anti-tumor agents against CRC.

**Conclusions:**

Inhibition of autophagy enhances synergistic effects of Sal and anti-tumor agents against colorectal cancer. This study provides experimental evidence and theoretical reference for improvement of a novel chemotherapy treatment protocol.

## Background

Although recent advances in diagnostic, surgical, and therapeutic techniques, colorectal cancer (CRC) causes high morbidity and mortality with approximately 62.7% of the 5-year survival rate worldwide [1, 2]. Chemotherapy remains one of the most common treatments for colon cancer, specifically for patients with the recurrent and metastatic diseases, to prolong survival time [1]. Currently, researchers attempt to add auxiliary agents to create additive or synergistic effects on standard treatment regimens. It’s long been known that numerous plant extracts possess potently anti-proliferative and pro-apoptotic effects against diverse cancer cells, and the intake is associated with reduced incidence and slow development of cancers [3]. However the effectiveness of using chemotherapeutic agents and plant extracts in combination is contradictory in cancer therapy in different studies. Herbal agent MB-6, which is derived from fermented soybean, green tea, grape seed and curcumin extracts, increase the effectiveness 5-fluoracil-based chemotherapy in CRC [4]. In addition, herb extract Tanshinone IIA improves the sensibility of chemotherapeutics for non-small cell lung cancer with fewer side effects, which may be a promising sensitizers for chemotherapy drugs [5]. However, evidence is increasing that some botanical extracts induce generation of autophagy of cancer cells, which impairs the toxicity of chemotherapeutic agents [6].

Autophagy is an adaptive response to nutrient deprivation and environmental stimulus, which can occur in both normal and cancer cells. It involves the lysosomal degradation of cellular components such as misfolded proteins or damaged organelles, to remove dysfunctional cytoplasmic constituents and recycle basic molecular building blocks [7]. The process not only maintains metabolic homeostasis, but also protects against cells death under adverse conditions. At present, autophagy has become an important anticancer target in chemotherapy, because autophagy is associated with increased chemo-resistance of cancer cells, responsible for consequent therapeutic failure [8]. In spite of their anti-proliferative effect, many plant extracts trigger the generation of autophagy [7, 9]. Autophagy is regulated by complicated signaling pathways of AMPK/mTOR, NF-κB, TGF-β, JAK2/STAT3 and so on [10–13]. Currently, the mechanism underlying the autophagy induced by botanical extracts is not well-understood.

Salidroside (Sal) is one of the major phenylpropanoid glycosides in *Rhodiola rosea L*, a medicinally important plant mainly found in the alpine area of China [14]. Previous reports have manifested that Sal administration inhibits the growth of human cancers, including CRC, breast cancer, lung cancer and renal cell carcinoma in vitro and in vivo [14–16]. This study was aimed to investigate the effectiveness of combined use of Sal with anti-tumor agents against CRC cells and moreover to evaluate the potential role of autophagy in the combined therapy.

## Methods

### Cell culture and treatment

CRC cell line was obtained from the American Type Culture Collection (ATCC CCL-247™, Manassas, VA, USA). HCT-116 cells were cultured in Dulbecco’s modified Eagle’s medium (Gibco-BRL, Grand Island, NY, USA) supplemented with 10% fetal bovine serum (Invitrogen, Life Technologies, Carlsbad, CA, USA) in a 37 °C incubator with 5% CO_2_.

Sal was purchased from Sigma-Aldrich (No. 43866-25MG; St Louis, MO, USA). HCT-116 cells in the logarithmic phase were treated with Sal (0, 0.5, 1 or 2 μg/mL) for different time periods to characterize the growth inhibitory effects. Besides, HCT-116 cells were subjected to antitumor drugs including oxaliplatin (OXA; NO. O9512), 5-fluorouracil (5-FU; NO. 04541) and Doxorubicin (ADM; NO. D1515; Sigma-Aldrich) to determine their IC25 values. Furthermore, HCT-116 cells were treated with Sal and antitumor drugs together to investigate their synergistic effects. Compound C and 3-Methyladenine (3-MA) were purchased from Selleck (Houston, Texas, USA). Their were added to cells to block AMPK signaling and autophagy, respectively.

### Cell viability assay

HCT-116 cell viability was evaluated using CCK-8 Detection Kit (Beyotime Institute of Biotechnology, Shanghai, China). HCT-116 cells were seeded into 96-well plates and subjected to indicated treatments. The cells were further maintained in 90 μl of culture medium plus 10 μl CCK-8 reagents per well for 1 h. The optical density at 490 nm (OD 490 nm) in each well was determined by an enzyme immunoassay analyzer (Bio-Tek ELX-800; Winooski, VT, USA).

### Cell apoptosis rate assay

The cell apoptosis rate was assessed using an Annexin V-FITC/PI Apoptosis Detection Kit (Beyotime Institute of Biotechnology) according to the manufacturer’s instructions. The HCT-116 cells were double-stained with Annexin V-FITC and propidium iodide in the dark, and then analyzed with a dual laser flow cytometer (Becton Dickinson, San Jose, CA, USA).

### Immunofluorescent (IF) assay

The HCT-116 cells were fixed with 4% paraformaldehyde for 15 min and blocked with PBS containing 0.3% Triton X-100/5% BSA (*w*/*v*) for 1 h at room temperature, before the incubation with antibody specific for LC3B (1:500, ab48394; Abcam, Cambridge, UK). Incubation with the secondary fluorescent-labeled antibody (Alexa Fluor 488, Invitrogen) was performed in the dark prior to the microscopic analysis (LeicaTCS-SP5 microscopy, Leica instrument co., LTD, Beijing, China).

### Western blotting

Total cell extracts were prepared using ice-cold lysis buffer (Sigma-Aldrich) containing protease inhibitor cocktail (Sigma-Aldrich). Proteins (20 μg/lane) were separated by SDS-polyacrylamide gel electrophoresis (10–15% gels) and transferred onto nitrocellulose membranes (Sigma-Aldrich). Membranes were blocked in 5% non-fat milk in TBS/0.1% Tween 20 for 2 h prior to immunoblotting overnight with antibodies against LC3B (1:500, ab48394; Abcam), phospho (p)-AMPKα (1:500; #2535, Cell Signaling Technology, Inc., Shanghai, China), p-mTOR (1:1000; #2971, Cell Signaling Technology), p-NF-κB (p65) (1:500; sc-166,748, Santa Cruz Biotechnology, Inc., Dallas, TX, USA), TGFβ1 (1:1000; ab31013, Abcam), p-JAK2 (1:1000; ab195055, Abcam), p-STAT3 (1:800; ab30647, Abcam), Beclin-1(1:500; ab55878, Abcam) and β-actin (1:1000, Santa Cruz Biotechnologies). Incubation with the secondary fluorescent-labeled antibody (Alexa Fluor 488) was performed for 2 h at room temperature in the dark. The proteins were visualized by enhanced chemiluminescence (Amersham Bio-sciences, NJ, USA).

### The knockdown of AMPKα

Small interference RNA (siRNA) targeting AMPKα (siRNA-AMPKα) was synthesized by GenePharma Co., Ltd. (Shanghai, China). The double-stranded sequences were: 5’-AUUCAUGUGUGCAUCAAGCTT-3′; 5’-GCUUGAUGCACACAUGAAUTT-3′. siRNA-AMPKα was transfected into HCT-116 cells using Lipofectamine™ 2000 (Invitrogen Life Technologies), according to the manufacturer’s instructions. Transfection efficiency was routinely 85 to 90%, as determined by transfection of enhanced green fluorescent protein reporter plasmid.

### Statistical analysis

For statistical analysis the Student’s t test was applied (SPSS13.0 software; Chicago, IL, USA). The results are presented as the mean ± S.E. of three independent experiments. *p* values of <0.05 was considered as significant and marked with an *asterisk*.

## Results

### Effectiveness of combined use of Sal with anti-tumor drugs against HCT-116 cells

**Fig. 1:**
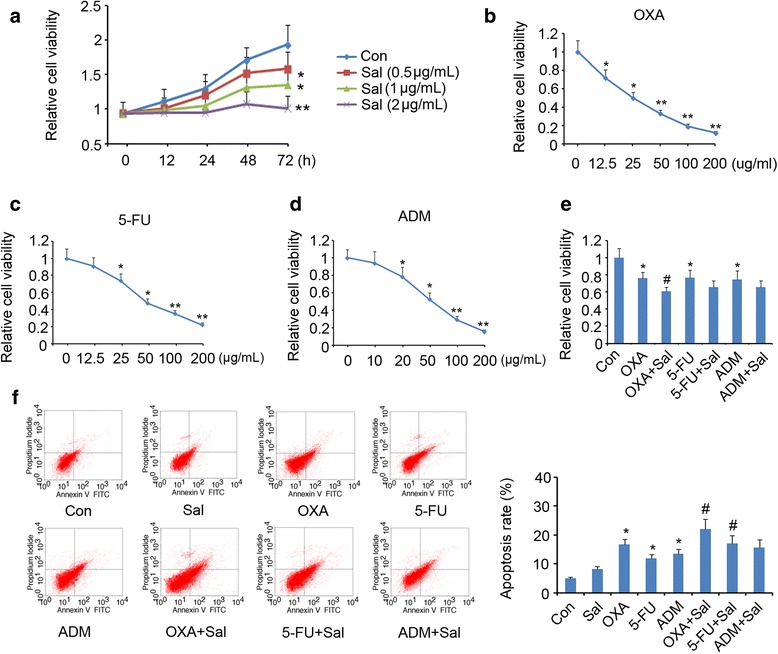
The effectiveness of Sal alone and in combination with anti-tumor agents against CRC cells. **a** HCT-116 cells were exposed to doses of Sal (0, 0.5, 1 and 2 μg/mL) for different time periods, followed by cell viability test. HCT-116 cells were subjected to doses of antitumor drugs including **b** OXA, **c** 5-FU and **d** ADM, followed by cell viability test. *P < 0.05 and **P < 0.01 vs. control. Evaluation of cell viability (**e**) and apoptosis rate (**f**) after the cells were treated with Sal and anti-tumor agents, alone and in combination. *P < 0.05 and **P < 0.01 vs. control; ^*#*^ *P* < 0.05 and ^*##*^ *P* < 0.01 indicated that anti-tumor agents (OXA, 5-FU and ADM) vs. corresponding anti-tumor agents + Sal. Sal: Salidroside; OXA: oxaliplatin; 5-FU: 5-fluorouracil; ADM: Doxorubicin

This study initially characterized the growth inhibitory effect of Sal on HCT-116 cells. HCT-116 cells were exposed to Sal (0, 0.5, 1 or 2 μg/mL) for different time periods. As shown in Fig. 1a, adding 0.5 and 1 μg/mL Sal for 72 h inhibited viability of HCT-116 cells (*p* < 0.05). 2 μg/mL Sal caused more effective inhibition of the cell viability than 0.5 and 1 μg/mL Sal (*p* < 0.01 vs. control, at 72 h).

HCT-116 cells were subjected to various concentrations of antitumor drugs including OXA, 5-FU and ADM to determine their IC25 values. The growth curves of HCT-116 cells after exposing to OXA, 5-FU and ADM were shown in Fig. 1b, c and d respectively. HCT-116 cell viability was decreased by 25% by 12.93 μg/mL OXA, 25.75 μg/mL 5-FU or 19.68 μg/mL ADM.

Treatment with 1 μg/mL Sal and 12.93 μg/mL OXA in combination showed improved effect on the inhibition of the cell viability compared to treatment with 12.93 μg/mL OXA alone (*p* < 0.05, Fig. 1e). But, inhibitory effects of 5-FU (25.75 μg/mL) and ADM (19.68 μg/mL) were only moderately enhanced by 1 μg/mL Sal.

Apoptosis assay showed that treatment with 1 μg/mL Sal for 72 h did not decrease apoptosis rate of HCT-116 cells significantly, but the apoptosis rate was reduced by OXA, 5-FU and ADM at concentrations of 12.93 μg/mL, 25.75 μg/mL and 19.68 μg/mL, respectively (*p* < 0.05, Fig. 1f). 1 μg/mL Sal conferred promoted effect on the apoptosis induced by OXA and 5-FU (*p* < 0.05), but only marginally increased the apoptosis induced by not by ADM.

### Sal promoted autophagy of HCT-116 cells through activating AMPK signaling

**Fig. 2:**
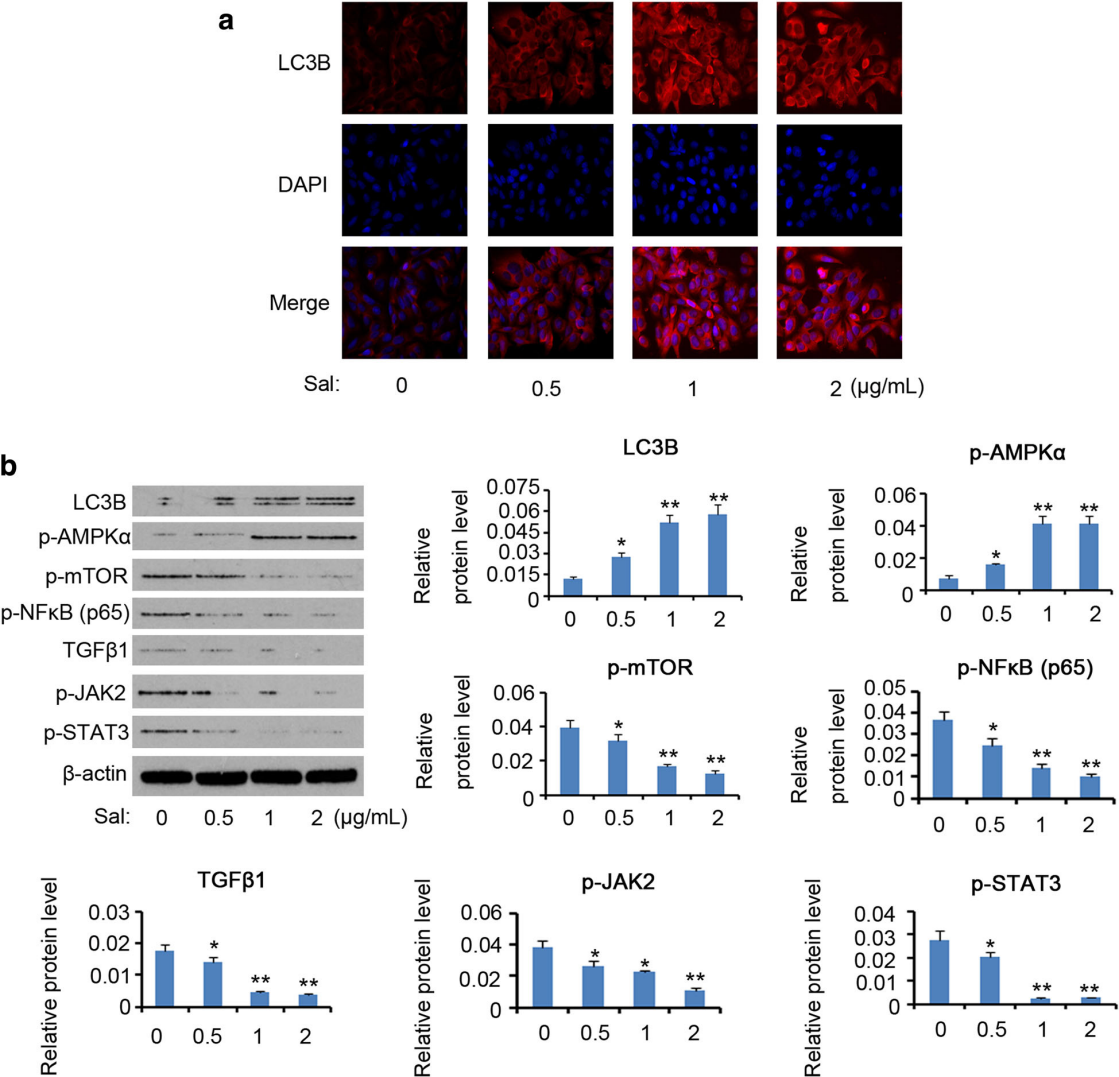
Signaling involves Sal regulating autophagy of HCT-116 cells. HCT-116 cells were exposed to doses of Sal (0, 0.5, 1 and 2 μg/mL) for 72 h. **a** IF assay showed LC3B expression. **b** Western blot assay detected expression of indicated proteins. *P < 0.05 and **P < 0.01 vs. control. Sal: Salidroside

To determine whether Sal promotes autophagy of HCT-116 cells, we examined expression levels of autophagic biomarkers following the treatment with Sal. In IF assay, fluorescence intensity of LC3B (microtubule-associated protein 1 light chain 3B), a well-known autophagic biomarker, was increased by Sal in a dose-dependent manner (Fig. 2a). Western blot measurement further confirmed that Sal forced LC3B expression in HCT-116 cells (Fig. 2b). To get insight into the mechanism underlying the autophagy triggered by Sal, this study detected expression of signaling molecules that control autophagy process. Sal dose-dependently increased expression of p-AMPK**,** but reduced p-mTOR, p-NF-κB (p65), TGFβ1, p-JAK2 and p-STAT3 expression. Previous literature represents that autophagy associates activation of AMPK**/**mTOR, NF-κB, TGFβ1 and JAK2/STAT3 cascades [10–13], thus it was likely that AMPK**/**mTOR signaling mediates Sal-induced autophagy.

**Fig. 3:**
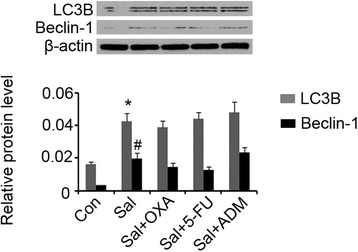
Sal increased expression of LC3B and Becline-1 alone or in combination with anti-tumor agents. HCT-116 cells were exposed to 1 μg/mL Sal alone or in combination with 12.93 μg/mL OXA, 25.75 μg/mL 5-FU or 19.68 μg/mL ADM for 72 h. Western blot assay detected expression of indicated proteins. *P < 0.05 and **P < 0.01 vs. control. Sal: Salidroside; OXA: oxaliplatin; 5-FU: 5-fluorouracil; ADM: Doxorubicin

We further investigated the influence of combined use of Sal with anti-tumor agents on cell autophagy. 1 μg/mL Sal elevated expression of LC3B (*p* < 0.05) and Becline-1(*p* < 0.05), as shown in Fig. 3. However, anti-tumor agents including OXA, 5-FU and ADM did not significantly up-regulated LC3B and Becline-1.

**Fig. 4:**
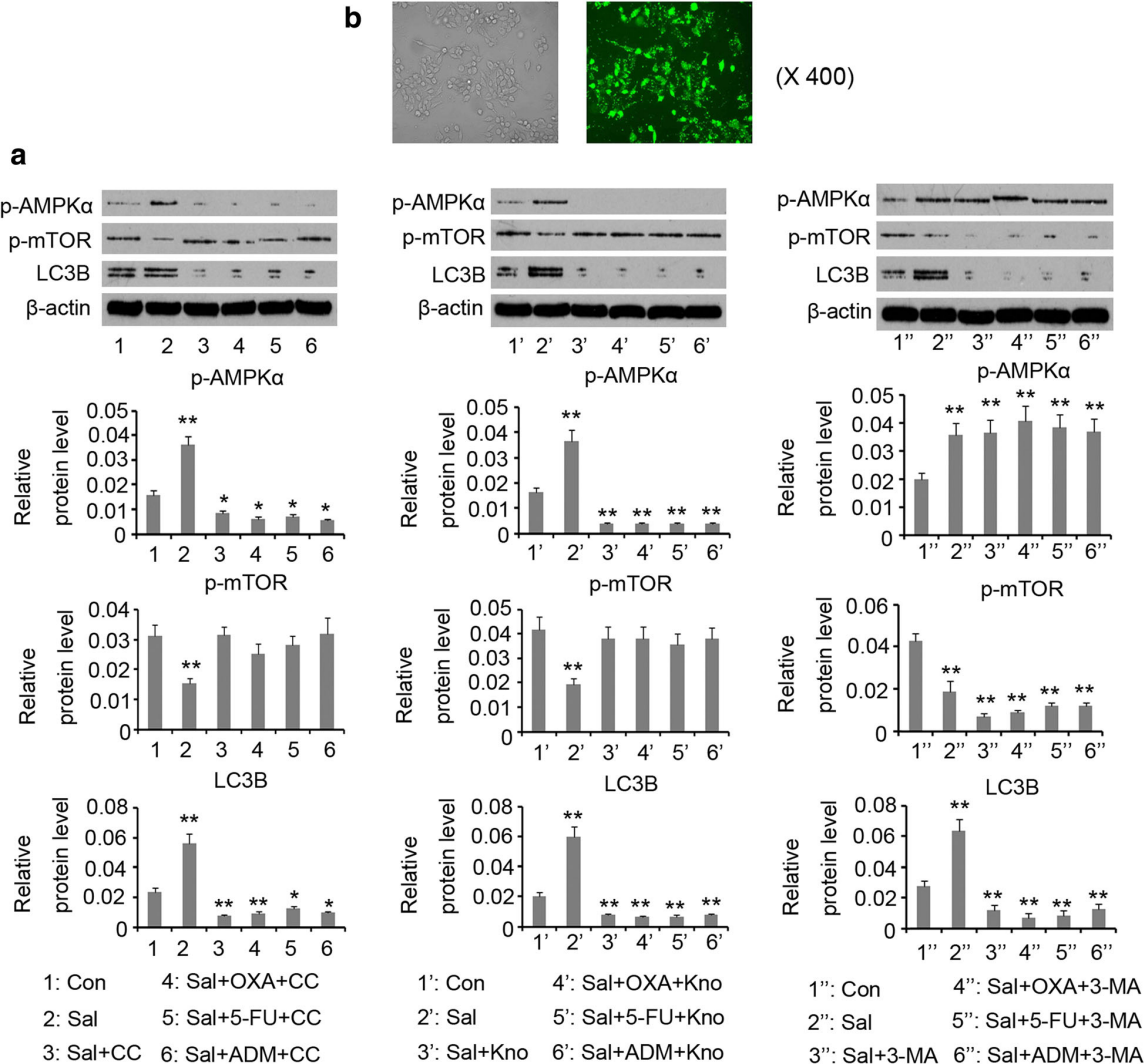
**a** Sal-induced autophagy was impaired by blockage of AMPK signaling or by 3-MA treatment. AMPK signaling was blocked via Compound C and AMPK knockdown. HCT-116 cells were exposed to 1 μg/mL Sal alone or in combination with 12.93 μg/mL OXA, 25.75 μg/mL 5-FU or 19.68 μg/mL ADM for 72 h. Western blot assay detected expression of indicated proteins. **b** The transfection efficiency of siRNA-AMPK was determined by the transfection of enhanced green fluorescent protein plasmids.*P < 0.05 and **P < 0.01 vs. control. Sal: Salidroside; OXA: oxaliplatin; 5-FU: 5-fluorouracil; ADM: Doxorubicin; Kno: AMPK knockdown; 3-MA: Methyladenine

To determine the role of AMPK**/**mTOR signal in Sal-induced autophagy, this study blocked this signal through adding AMPK inhibitor (Compound C) and knocking down AMPK via siRNA-AMPKα. 10 μM Compound C reversed the up-regulation of p-AMPK induced by Sal (*p* < 0.05 vs. control, Fig. 4a). In addition, co-treatment of the Compound C, Sal and anti-tumor agent (OXA, 5-FU or ADM) decreased p-AMPK protein level (*p* < 0.05 vs. control). Compound C restored p-mTOR level that was decreased by Sal. Sal increased LC3B expression (*p* < 0.05 vs. control), but treatment with Sal and Compound C in combination reversed LC3B expression (*p* < 0.01 vs. control). Adding OXA had modest effect on LC3B expression that was decreased by combined use of Sal and Compound C (*p* < 0.01 vs. control), whereas 5-FU and ADM to some degree restored LC3B expression (*p* < 0.05 vs. control). Transfection with siRNA-AMPKα was performed for AMPK knockdown. The transfection efficiency was routinely determined by transfection of enhanced green fluorescent protein reporter plasmid (Fig. 4b). AMPK knockdown before the treatment with Sal suppressed the p-AMPK and LC3B expression (*p* < 0.01 vs. control, Fig. 4a) and restored the decreased p-mTOR. 3-MA is commonly used to block the initiation of autophagy. 3-MA reversed the up-regulation of LC3B induced by Sal (*p* < 0.01 vs. control), but conferred modest effects on p-AMPK and p-mTOR protein levels.

### The effectiveness of combined use of Sal with anti-tumor drugs was increased by inhibiting AMPK signal and autophagy

**Fig. 5:**
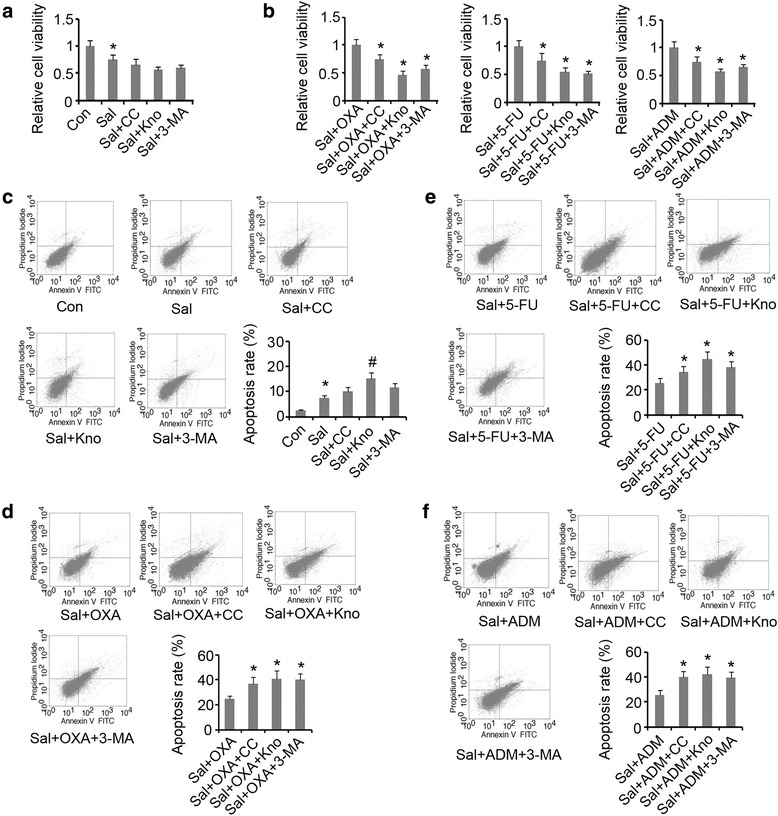
Inhibition of autophagy enhanced synergistic effects between Sal and anti-tumor agents against colorectal cancer. **a** Compound C, AMPK knockdown and 3-MA did not significantly influence the decreased cell viability by Sal. Sal group was compared with control (*P < 0.05); Sal + CC, Sal + Kno and Sal +3-MA groups were compared with Sal group (no significant difference between groups). **b** Inhibition of autophagy further decreased the viability of cells treated with Sal and anti-tumor agents. *P < 0.05 vs. control. **c** Apoptosis induced by Sal was enhanced by AMPK knockdown, but not by Compound C and 3-MA. *P < 0.05 vs. control and ^*#*^ *P* < 0.05 vs. Sal group. **d**, **e** and **f** Inhibition of autophagy further enhanced apoptosis induced by Sal and anti-tumor agents including OXA, 5-FU and ADM. *P < 0.05 vs. control. Sal: Salidroside; OXA: oxaliplatin; 5-FU: 5-fluorouracil; ADM: Doxorubicin; Kno: AMPK knockdown; 3-MA: Methyladenine

Compound C, AMPK knockdown and 3-MA marginally decreased viability of the HCT-116 cells treated with Sal (Fig. 5a). However, Compound C, AMPK knockdown and 3-MA dramatically reduced viability of the cells treated with Sal and anti-tumor drug (OXA, 5-FU or ADM) in combination (*p* < 0.05, Fig. 5b). Apoptosis rate of the HCT-116 cells treated with Sal was increased by AMPK knockdown (*p* < 0.05, Fig. 5c), but not by treatment with Compound C or 3-MA. Compound C, AMPK knockdown and 3-MA notably promoted apoptosis induced by combined use of Sal and anti-tumor drug (OXA, 5-FU or ADM) (*p* < 0.05, Fig. 5d–f).

## Discussion

Various plant extracts have been suggested to be used as auxiliary agents in chemotherapy due to their anti-proliferative and pro-apoptotic effects. However, combined use of chemotherapeutic agents and plant extracts is rather controversial in cancer therapy. That is because some plant extracts trigger the generation of autophagy of cancer cells while suppressing the proliferation [7, 9]. Autophagy confers protective effect against toxicity of anti-tumor agents, thus it, to some extent, inhibits apoptosis of cancerous cells and results in chemo-resistance [17]. Sal has been emerged as an important plant extracts for the treatments of cancer and age-related diseases [18]. Our data showed that Sal inhibited cell viability of CRC, but only exerted modest effect on the apoptosis at a moderate concentration (1 μg/mL). Sal showed synergistic effects with OXA against CRC, but the synergistic effect between Sal and ADM was not evident. The present study found that Sal induced generation of autophagy of HCT-116 cells. Intervention of autophagy via 3-MA notably promoted synergistic effects between Sal and anti-tumor drugs including OXA, 5-FU and ADM. This finding suggests that Sal-induced autophagy probably impaired the synergistic effects with anti-tumor agents against CRC.

Autophagy is an important cellular process in response to various stress conditions to maintain proper cell function and homeostasis through the degradation of damaged intracellular proteins and organelles and the recycling of amino acids, free fatty acids or nucleotides derived from the degradation process [7, 9]. Therefore, autophagy is generally recognized as a cell pro-survival mechanism, although excessive induction of autophagy becomes an alternative mechanism for programmed cell death when exposing to overwhelming or persistent stress [8, 19]. It has been suggested that targeting cytoprotective autophagy is a critical strategy to improve the anticancer activity of therapeutics [8]. Previous literature has demonstrated that sorafenib is able to activate autophagy, which confers a survival advantage to cancer cells and leads to sorafenib resistance. However, inhibiting autophagy promotes sorafenib-induced cell death and increase its anticancer effect [20]. In addition, inhibition of autophagy facilitates sensitivity of gefitinib and salinomycin in breast cancer and prostate cancer respectively [8, 21].

Autophagy is easily induced when cancer cells are exposed to some plant extracts, although the underlying mechanism is largely unknown. Autophagy is regulated by various signaling pathway, such as AMPK/mTOR, NF-κB, TGF-β and JAK2/STAT3 [10–13]. To shed light on molecular basis that mediates Sal-induced autophagy, this study examined their expression profiles following Sal induction. Except for the up-regulation of p-AMPK, p-mTOR, p-NF-κB (p65), TGF-β, p-JAK2 and p-STAT3 were down-regulated by Sal. Previous literature has represented that autophagy is positively correlated with the activation of AMPK**/**mTOR, NF-κB, TGFβ1 and JAK2/STAT3 cascades [10–13], thus Sal-induced autophagy may associate AMPK signaling. To verify this hypothesis, this study blocked AMPK signaling via adding the specific inhibitor Compound C and transfecting siRNA-AMPK. Both Compound C and AMPK knockdown reversed up-regulation of p-AMPK induced by Sal. The blockage of AMPK signaling is accompanied with reduced LC3B expression, suggesting that autophagy is inhibited. Therefore, it is suggested that AMPK signaling mediates autophagy of CRC cells induced by Sal.

The molecular mechanism by which AMPK regulates autophagy has been revealed in previous reports. AMPK regulating autophagy is dependent or independent of mTOR, through AMPK-mTOR-S6 K and AMPK-ULK1 signaling pathways, respectively [22, 23]. mTOR activity is negatively modulated by AMPK, but mTOR inhibition associates improved autophagy, that is the reason why mTOR selective inhibitor Rapamycin is usually used as autophagy agonist. Although the present study demonstrated that Sal-induced autophagy is dependant of AMPK signaling, the mechanism by which Sal activates AMPK is unclear. Previous reports have suggested that plant extracts disturbing intracellular anti−/oxidative homeostasis is likely an important reason for AMPK activation [6, 8, 9]. Oxidative species have been confirmed to participate in the regulation of cell signaling. Further study should be warranted to elucidate mechanism by which Sal activates AMPK.

**Fig. 6:**
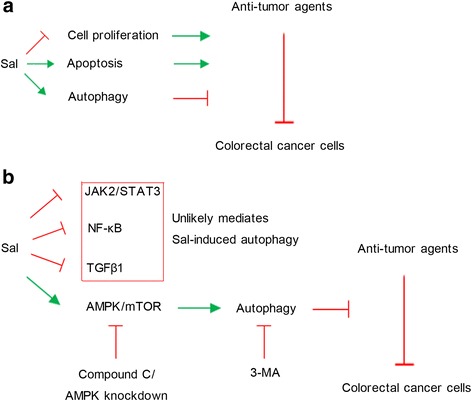
Autophagy impairs synergistic effects of Sal and anti-tumor agents against CRC. **a** Although Sal exerts anti-proliferative and pro-apoptotic effects, it triggered autophagy of CRC cells, which counteracts the anti-tumor effects of Sal alone or in combination with anti-tumor agents. **b** Sal up-regulated p-AMPK, but down-regulated p-mTOR, p-NF-κB (p65), TGF-β, p-JAK2 and p-STAT3 in CRC cells. It was demonstrated that autophagy induced by Sal mainly associates with AMPK activation. Blockage of AMPK signaling via compound C and AMPK knockdown or direct inhibition of autophagy via 3-MA increased effectiveness of combined use of Sal with anti-tumor agents against CRC, which suggests that autophagy impairs synergistic effects of Sal and anti-tumor agents

Previous study has demonstrated that Sal exerts anti-proliferative and pro-apoptotic effects against cancer cells [18]. However, the present study found that Sal also triggered autophagy of CRC cells, which counteracts the anti-tumor effects of Sal alone or in combination with anti-tumor agents (as shown in Fig. 6a). Treatment with Sal up-regulated p-AMPK, but down-regulated p-mTOR, p-NF-κB (p65), TGF-β, p-JAK2 and p-STAT3 in CRC cells. It was hypothesized that autophagy induced by Sal mainly associates with AMPK activation (as shown in Fig. 6b). This hypothesis was demonstrated by treatment with Compound C and by AMPK knockdown, as both of them inhibited the autophagy. The blockage of AMPK signaling or a direct inhibition of autophagy via 3-MA increased effectiveness of combined use of Sal with anti-tumor agents against CRC, which suggests that autophagy impairs synergistic effects of Sal and anti-tumor agents.

## Conclusions

Inhibition of autophagy enhances synergistic effects of Sal and anti-tumor agents against colorectal cancer. This study provides experimental evidence and theoretical reference for improvement of a novel chemotherapy treatment protocol.

## Abbreviations

5-FU: 5-fluorouracil
ADM: Doxorubicin
CRC: colorectal cancer
OXA: oxaliplatin
Sal: Salidroside
