# Correction to: Inhibition of autophagy enhances synergistic effects of Salidroside and anti-tumor agents against colorectal cancer

**DOI:** 10.1186/s12906-018-2120-1

**Published:** 2018-02-21

**Authors:** Hai Li, Chen Chen

**Affiliations:** 1 Department of Geriatrics, Tongji Hospital, Tongji Medical College, Huazhong University of Science and Technology, Wuhan, China; 2 Department of Breast and Thyroid Surgery, Union Hospital, Tongji Medical College, Huazhong University of Science and Technology, Wuhan, China

## Correction

The authors of this article [1] would like the funding and acknowledgements to be disregarded.
